# Structure of FIV capsid C-terminal domain demonstrates lentiviral evasion of genetic fragility by coevolved substitutions

**DOI:** 10.1038/srep24957

**Published:** 2016-04-22

**Authors:** Aya Khwaja, Meytal Galilee, Ailie Marx, Akram Alian

**Affiliations:** 1 Faculty of Biology, Technion – Israel Institute of Technology, Haifa 320003, Israel

## Abstract

Viruses use a strategy of high mutational rates to adapt to environmental and therapeutic pressures, circumventing the deleterious effects of random single-point mutations by coevolved compensatory mutations, which restore protein fold, function or interactions damaged by initial ones. This mechanism has been identified as contributing to drug resistance in the HIV-1 Gag polyprotein and especially its capsid proteolytic product, which forms the viral capsid core and plays multifaceted roles in the viral life cycle. Here, we determined the X-ray crystal structure of C-terminal domain of the feline immunodeficiency virus (FIV) capsid and through interspecies analysis elucidate the structural basis of co-evolutionarily and spatially correlated substitutions in capsid sequences, which when otherwise uncoupled and individually substituted into HIV-1 capsid impair virion assembly and infectivity. The ability to circumvent the deleterious effects of single amino acid substitutions by cooperative secondary substitutions allows mutational flexibility that may afford viruses an important survival advantage. The potential of such interspecies structural analysis for preempting viral resistance by identifying such alternative but functionally equivalent patterns is discussed.

In the evolutionary host-virus arms race, interactions are based on an endless cycle of adaptations in which the virus necessarily evolves to manipulate and survive the hostile host environment and the host adapts to disrupt this manipulation. The human immunodeficiency virus (HIV) manages to escape eradication by drugs and immune responses mainly through a strategy of high turnover and extremely high mutational rate. Random mutations, however, rarely correlate directly to survival adaptations and more often have deleterious effects. Mutations can directly impair enzymatic activity or perturb interfaces central to intrinsic folding or extrinsic interactions essential for crafting protein assemblies of virus and viral-host complexes. Auxiliary mutations, which induce compensatory structural and functional changes, can stabilize a mutant protein facilitating its persistence in an evolved virus strain (reviewed in^1^). Essentially, coevolved second-site compensatory mutations repair the protein fold and/or enzymatic activity, or fix protein interfaces vital for interactions with other proteins^2^,^3^. The latter strategy may be achievable via complementary mutations in the binding partner^3^ or through interactions with alternative partners, which are functionally equivalent and redundantly available, resulting in rerouting-resistance^4^.

Positions of amino acid pairs evolving in a correlated manner have long been proposed to prescribe protein structure and function, and such correlation rules have been found sufficient to describe protein fold and guide the design of artificial sequences that consequently fold into native structures^2^. Co-evolution guided structure prediction has neatly been demonstrated in a recent study accurately modeling novel protein structures using distance restraints between amino acid pairs as predicted from co-evolutionary patterns^5^, a method that can further be enhanced upon combining the strong correlations of amino acid pairs with their weak correlations at the genetic codon-level^6^.

In addition to providing insight into the basic relationship between amino acid sequence and protein fold, identifying and characterizing correlated substitutions in naturally diverse and coevolved resistant viruses is imperative to drug and vaccine development^3^. One of the promising therapeutic targets in HIV-1 biology is the Gag polyprotein and especially its capsid (CA) proteolytic product, which forms the viral capsid core and plays multifaceted fundamental roles in the viral life cycle^7^,^8^,^9^,^10^. Comprehensive analyses of HIV-1 Gag sequences, examining patterns of variability for such correlation rules, have indeed identified numerous coevolved pairs of linked mutations contributing to drug resistance^7^,^8^,^11^,^12^,^13^,^14^ and have been attributed either to natural virus polymorphism^7^,^11^ or drug-challenges^15^,^16^,^17^. Although one study demonstrated that HIV-1 CA is an extremely genetically fragile protein, with 70% of the 135 tested single point mutations (covering 44% of CA sequence) yielding replication defective viruses^18^, another showed that mutating the most conserved residues in HIV-1 CA does not strongly associate with the highest fitness costs^19^. These apparently contradictory results can be consolidated by considering conserved functional coupling of mutations since focusing on the specific identities of conserved residues or individual mutations, rather than coevolved patterns, could suggest misleading fragility.

To extend our understanding of the functional and spatial correlations of coevolved substitutions, we investigated the primary and tertiary structures of CA from a non-primate lentivirus, the feline immunodeficiency virus (FIV). FIV is the lentivirus that most closely parallels HIV-1 in replication biology and pathogenesis, and is the only non-primate model in which the lentivirus induces an AIDS-like syndrome in its natural host. This makes FIV an attractive small animal model for anti-AIDS vaccine and drug research^20^,^21^. While proteins from the feline lentivirus share an abundance of structural and functional commonalities with HIV-1 (reviewed in^22^), their amino acid sequences highly diverge. Therefore, pinpointing interspecies subtleties can highlight crucially conserved patterns for drug and vaccine targeting, and more importantly, can uncover conceivably latent escape patterns accessible to challenged HIV-1. Here we show the structural basis of co-evolutionarily and spatially correlated substitutions in non-primate CA sequences, which when otherwise uncoupled and individually substituted into HIV-1 CA impair virion assembly and infectivity.

## Results and Discussion

**Figure 1:**
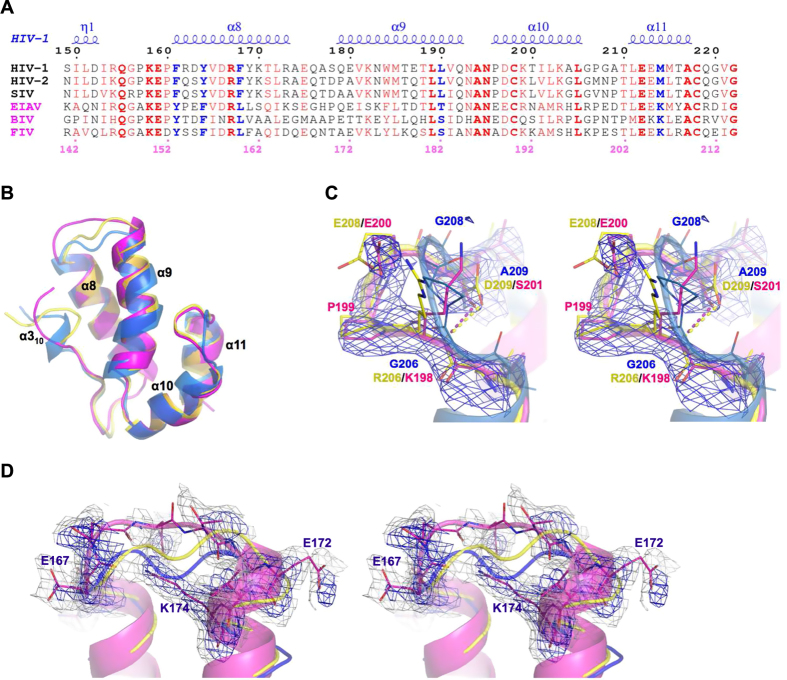
Preservation of FIV CA^CTD^ overall structure. (**A**) Sequence alignment of CA^CTD^. Top numbering and secondary structure are shown for HIV-1 (4XFX) and bottom numbering for FIV. Identical residues are shown in bold red font and conserved equivalents in red font. Bold blue font indicates correlated sites that are different between primate (black titles) and non-primate (pink titles) lentiviruses. (**B**) Superposition of CA^CTD^ of FIV (magenta), HIV-1 (blue, 4XFY) and EIAV (yellow, 1EIA). *F*_*O*_*-F*_*C*_ map (blue mesh, 2.5 σ) calculated after omitting corresponding fragments of hook-like residues 197–202 (**C**) or loop (residues 166–174) connecting α-8/α-9 (gray mesh represents *2F*_*O*_*-F*_*C*_ map at 1.0 σ) (**D**). Panels (**C**) and (**D**) are in walleye stereo view.

Capsid (CA) proteins from different retroviruses have remarkably low sequence identity^23^,^24^. So divergent are the sequences that even amino acids demonstrated as critical in a particular retrovirus are non-conserved in another. For example, in HIV-1 CA the Y164F or G208E mutations attenuate virion production and infectivity^18^. Despite this essentiality for the function of HIV-1 CA, these amino acids are not individually conserved and CA from feline immunodeficiency virus (FIV) inherently harbors F and E at these positions respectively (Fig. 1A). Nevertheless, the α-helical fold of the CA protein and capsid-core assemblies are highly conserved across the various retroviruses including HIV-1, equine infectious anemia virus (EIAV), human T-cell leukemia virus type I (HTLV) and Rous sarcoma virus (RSV) (reviewed in^25^). There appears to be a strong selective pressure that necessitates maintenance of a specific functional structure even during the mutation and evolution of CA encoding sequences^10^,^23^,^26^. Indeed, a more holistic sequence comparison demonstrates the significance of coevolved substitutions and resolves the conundrum of the strict conservation of structure and function despite considerable variability in amino acid sequences. A simple sequence analysis immediately highlights that the Y164 mentioned above forms part of a conserved amino acid pair F/Y in primate viruses (HIV-1 F161/Y164) that is switched to Y/F in non-primate viruses (Fig. 1A). This suggests that the deleterious action of the single Y164F mutation in HIV-1 CA might be rescued by the acquisition of a second-site mutation, F161Y, which would restore the conserved amino acid set. To probe for such structurally essential and potentially correlated pairs in the lentiviral CA, we determined the structure of the C-terminal domain (CTD) of FIV CA (CA^CTD^), which naturally evolved to cope with substitutions individually shown deleterious to HIV-1 CA, and compared it to other non-primate and primate lentiviral CA structures available in the Protein Data Bank. Importantly, the CTD segment of CA, unlike the N-terminal domain (NTD), has been reported as biologically equivalent and functionally transferable between primate and non-primate lentiviruses^23^, making the interspecies comparison between FIV and HIV-1 physiologically relevant and especially convincing.

### The highly preserved FIV CA^CTD^ fold reveals only minor structural subtleties

Unsurprisingly, FIV CA encoded an absolutely conserved CTD fold comprising the characteristic four α-helical bundle (α-8 to α-11), which perfectly superimposes to CA of EIAV (0.55 Å RMSD) and HIV-1 (1.14 Å RMSD in average of several structures) (Fig. 1B).

Along with maintaining the highly conserved fold, the structure of FIV CA^CTD^ also retains the delicately tuned plasticity of CA α-helices and connecting-loops, which drive and support the dynamic structural changes that CA undergoes during the viral lifecycle. In the FIV CA^CTD^ structure, the flexible 3_10_-helix adopts a rather extended conformation similar to a previous NMR observation in tubular assemblies of HIV-1 CA^27^ (Fig. 1B). Since this 3_10_-helix is contained within the inter-domain linker connecting CTD and NTD domains, its dynamic nature has been anticipated to allow the flexible reorganization of the two domains during CA assembly^27^. The loop connecting α-10 and α-11 forms a distinct hook-like structure in FIV CA^CTD^, a feature that is not seen in the HIV-1 CA structures but is superimposable to that of the EIAV CA structure (Fig. 1B,C). This hook-like configuration appears necessary to accommodate the larger Glu, Lys and Arg side chains in non-primate lentiviruses as compared to the conserved Gly in primate viruses (discussed below). Another elastic connector is the flexible loop connecting α-8 and α-9, which was poorly resolved in the FIV CA^CTD^ structure and could not be modeled exactly according to structures from HIV-1 or EIAV as these would clash with the well-structured K174 of FIV CA^CTD^ (Fig. 1D). This loop has indeed been shown to display a wide range of conformations in reported CA structures, depending on the arrangement of the dimeric interfaces, and is missing in some crystal structures^28^ and in the solid-state NMR analysis of tubular CA^27^. Flexibility of this loop correlates with the crucial pliability of α-9, which in the FIV CA^CTD^ structure was best superimposed to α-9 of HIV-1 CA in the CAI-inhibitor bound (PDB: 2BUO^29^) and the dehydrated crystal (PDB: 4XFY^30^) states (Fig. 1B). A disulfide bridge, conceivably important for CA flexibility and formed between two conserved lentiviral Cys residues (190 and 210 in FIV), was reduced in a similar manner to structures of HIV-1 CA bound to CAI^29^ and domain-swapped^24^.

### A novel dimeric interface in the FIV CA^CTD^ structure mimics the binding of the CAI inhibitor helical-peptide

Flexibility of CA is most appreciated in playing a pivotal role during conformational transitions, which are triggered upon proteolytic maturation of the viral Gag-polypeptide^31^,^32^ and result in the trimeric CTD–CTD interface unique for mature capsid formation and stability^30^,^32^. Distinct CA interacting interfaces have been implicated at different stages of Gag maturation including NTD α-4 packing against a hydrophobic pocket, which is formed by α-8 and α-9 in the CTD^30^,^33^. This hydrophobic pocket was previously shown to bind small molecules^30^ and CAI peptide^29^ inhibitors, and it has been proposed to interact with host cofactors^10^. Mutational analysis of pocket-residues suggested that CTD–CTD dimerization is very sensitive to the detailed contacts within this conserved groove and underscored an allosteric role in regulating CA reorganization^33^.

**Figure 2:**
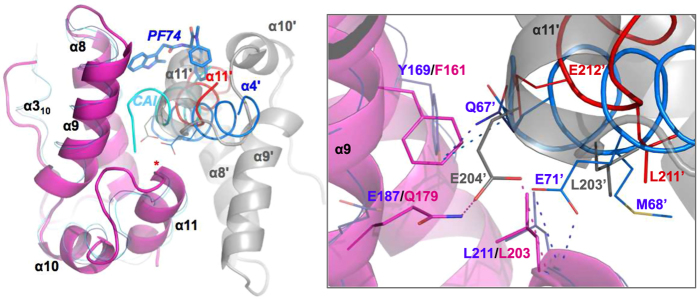
FIV CA^CTD^ crystal packing. FIV CA^CTD^ dimer (magenta and gray) compared to HIV-1 CA bound to CAI helical-peptide (cyan ribbon, 2BUO), symmetry NTD α-4 (blue ribbon, 4XFZ) or PF74 inhibitor (blue sticks, 4XFZ). Symmetry HIV E71′ and FIV E204′ are shown in blue and gray lines, respectively. α-11′ from a symmetry mate of HIV Y169A mutant is shown (red ribbon, 3DS2). Red asterisk indicate position of HIV L211 (FIV L203). Inset: close up of the core around the red asterisk. Residues of HIV and FIV are shown in lines (symmetry mates denoted with a prime-symbol). Dashed magenta (FIV) and blue (HIV) lines denote 2.6 – 4.5 Å distances. Two conformations for Q67′, native (blue) and modeled (dark blue), are shown. Residue labels are colored in accordance to structure (HIV: blue, FIV: magenta, EIAV: yellow).

The asymmetric unit of the FIV CA^CTD^ crystal contains two CTD molecules packed through a novel dimeric interface that docks the α-11′ symmetry mate (denoted with the prime symbol) into this conserved pocket resembling the binding of the CAI helical-peptide and PF74 small molecule (Fig. 2). α-11′ docking at this groove also parallels the packing of α-11′ from symmetry related molecules in the crystal packing of two HIV-1 CA mutants, Y169A and L211S (PDB: 3DS2 and 3DPH^33^), and comparison with these structures demonstrates the spatial superposition of two conserved residues, Glu (HIV-1 E212′ and FIV E204′) and Leu (HIV-1 L211′ and FIV L203′) (Fig. 2 and inset, red helix). Indeed, a E212A mutation in HIV-1, which would disturb such α-11 docking reduces infectivity by 3-fold^25^, implicating a potential biologic relevance of such docking, perhaps transient, during capsid maturation or assembly.

Walling the groove are FIV F161 (HIV-1 Y169) and L203 (HIV-1 L211) that appear essential for packing the α-11 from the adjacent monomer (Fig. 2, inset). These residues are indeed conserved in most retroviruses and while the overall intrinsic structures of HIV-1 Y169A and L211A/S CA^CTD^ mutants were not altered, cone-shaped cores and virus infectivity were completely lost, suggesting that these positions are critical for CA reorganization and assembly of mature-like particles^33^. Further, HIV-1 Y169 and L211 were also implicated in the docking of α-4 of NTD to this CTD groove (Fig. 2, blue helix). This packing of α-4 places HIV-1 vital and conserved E71′ (and Q67′)^25^ in a close spatial resemblance to the conserved E204′ of FIV α-11, mimicking comparable contacts with the backbone amide of HIV-1 L211 (FIV L203) from adjacent CA monomer (Fig. 2, inset). Therefore, the novel crystal packing of FIV CA^CTD^ molecules via the binding groove of CTD from one monomer and α-11′ of the adjacent one, while resembling reported interactions of HIV-1 CA subunits, suggests that the subtle allosteric groove of FIV CA^CTD^ is also preserved despite the different amino acid composition.

### FIV CA^CTD^ coevolved-substitutions preserve the functional fold despite sequence divergence

The high sequence homology (~40% identity) between FIV and HIV-1 CA^CTD^ sufficiently encoded for a highly preserved structure. Still, 70% of the 135 tested single point mutations in HIV-1 CA yielded defective viruses^18^, indicating that the divergent sequences comprise coupled substitutions that compensate for the sequence alterations in delicately tuning and preserving the functional fold of CA. To uncover such compensatory correlation rules, we analyzed the divergent sets of structurally correlated amino acids at the inter- and intra-molecular interfaces facilitating such delicate structural preservation. The amino acid sets examined here were those that can directly modulate intrinsic stability, folding and self-assembly of the CA protein. Amino acid sets that can alter CA patterns in exploiting different cellular cofactors and alternative pathways were beyond the scope of this study.

**Figure 3:**
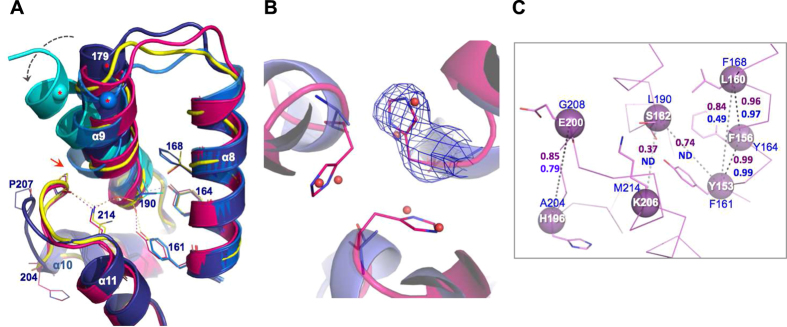
Structural basis of correlated substitutions. (**A**) Superposition of CA^CTD^ of FIV (magenta), EIAV (yellow, 1EIA), HIV-1 native (dark blue, 4XFX), dehydrated (blue, 4XFY) and domain-swapped (cyan, 2ONT) forms. HIV-1 residue numbering is shown. Dynamic N-terminus of α-9 (spheres with red asterisks: Cα of E179) is highlighted with a dashed gray arrow. Red arrow stresses steric hindrance between HIV P207 and α-9 bulge. Interactions are shown in dashed lines colored yellow (EIAV), blue (HIV) or magenta (FIV). Residue 190 denotes the static C-terminus base of α-9. (**B**) Trimeric CA interface of FIV (magenta) modeled per native HIV-1 structure (dark blue, 4XFX). HIV A204 (blue lines) and water molecules (red spheres) and FIV H196 (magenta lines) are shown. *F*_*O*_*-F*_*C*_ map (blue mesh, 2.5 σ) calculated after omitting H196 of FIV CA. (**C**) Correlated residues of FIV CA (magenta ribbon) are presented in magenta spheres (Cα) and side chains (lines). Residue numbering of FIV (white font) and HIV-1 (blue font) are shown. Correlated pairs are coupled with dashed lines of black (high score) or gray (weaker score) colors and the Gremlin’s probability score^5^ is shown in magenta (FIV) and blue (HIV-1) font. ND: not detected.

At the core of HIV-1 CA^CTD^ is a conserved Y164. Mutation to Phe, naturally present at this position in other retroviruses^24^, causes a 36% reduction in HIV-1 single-cycle infectivity and a 69% decrease in spreading fitness^18^, suggesting a functional role of the Y164 hydroxyl oxygen. Indeed, structural analysis of the available HIV-1 CA structures reveals a conserved interaction (2.5 Å) between this oxygen and the backbone amide of residue 190, an interaction that appears to pin down the C-terminal end of α-9 while leaving its N-terminal end extremely flexible (Fig. 3A). In order to maintain this apparently essential structural feature in the presence of Phe rather than Tyr, non-primate lentiviruses have apparently coevolved the residue at 161 (F161 in HIV-1) as Tyr so preserving (in a flipped direction) the F161/Y164 pattern of HIV-1 as Y153/F156 (FIV CA) (Fig. 1A). This configuration both preserves the pinning interaction between Tyr and the C-terminal end of α-9 (Fig. 3A) and maintains the hydrophobic core features. Therefore, whilst the F/Y pair is imperative for structural integrity, the relative position of either residue is apparently interchangeable.

Indeed, comparison of primate and non-primate CA^CTD^ amino acid sequences highlights a sequence pattern, which extends beyond this F/Y or Y/F pair, to include F/Y/F/L/M in primate viruses and Y/F/L/S(T)/K in FIV and other non-primate viruses (Fig. 1A). Switching the L190/M214 hydrophobic packing pair of primate viruses into the S(T)/K polar pair in non-primate viruses creates a potential hydrogen bond in the latter structure (Fig. 3A). Creation of such a polar interaction apparently requires the presence of both S(T) and K since a single substitution to L190S or M214L in HIV-1 CA resulted in either non-viable viruses or viruses with diminished virus viability and infectivity^18^. Therefore, the FIV inherent S182 (T190 in EIAV), at this HIV-1 190 position (Fig. 1A), may have been tolerated in the non-primate viruses by the co-acquisition of the compensatory Lys partner (K206 in FIV, and K214 in EIAV, instead of HIV-1 M214) (Fig. 3A). The stabilizing effect of the HIV-1 L/M packing or FIV S/K interaction appears crucial in the accurate positioning and pinning of the α-9 C-terminal base in a similar manner to the F/Y or Y/F pair discussed above.

FIV K206 also interacts (2.7 Å) with the carbonyl oxygen of the conserved FIV P199 (HIV-1 P207) stabilizing the above-described hook-like structure of the loop connecting α-10 and α-11. In HIV-1, this loop, which has been shown to mediate the trimeric interface^30^ and significantly change conformation^27^ during CA assembly, harbors a crucial G208 residue and a G208E mutation resulted in diminished HIV-1 infectivity and spreading fitness^18^. FIV CA inherently possesses E200 (E208 in EIAV) at this position, however the detrimental effect of HIV-1 G208E appears to have been compensated by the coevolution of a polar side-chain in FIV, S201 (D209 in EIAV), which substitutes HIV-1 A209 and interacts (2.9 Å) with the backbone amide of residue 198 in FIV (206 of HIV-1/EIAV), which forms the hook-like structure accommodating and “pulling-back” the larger E200 residue (Fig. 1C). Indeed, a polar side-chain substitution with A209T in HIV-1 retained wild type infectivity (108%) and spreading fitness while substitution with a nonpolar side-chain, A209V, retained only 72% infectivity^18^.

The smaller non-primate L160 (Y/F/**L**/S(T)/K/H) substituting the primate F168 (F/Y/**F**/L/M/A) correlates better with the hook-like structure since F168 in HIV-1 causes a bulge in the middle of α-9 that would conceivably clash with conserved P207 in a non-primate hook-like structure(Fig. 3A, red arrow).

Similarly, the overall structural conservation at the delicate trimeric 3-fold interface also conceals the significant sequence variations and corresponding adaptations. The HIV-1 A204 is substituted with a bulkier H196 in FIV CA (Fig. 1A). While hydrophobic substitutions of A204 to V/L retained HIV-1 infectivity and capsid stability, A204D replacement resulted in the production of non-infectious virions with unstable and abnormal cores^30^,^34^. Interestingly, two strategically structured water molecules, positioned near A204 at the trimeric interface of native CA (Fig. 3B), were lost upon crystal dehydration resulting in tighter packing of CA subunits^30^. The hydration layer, especially at the two- and threefold interfaces, has been proposed to strategically stabilize these variable interfaces and complement the flexible surface of CA^30^. Superposing the FIV structure to the trimeric interface of HIV-1 native CA structure (PDB: 4XFX) reveals the accurate placement of the FIV H196 imidazole ring onto the two waters from each HIV-1 CA monomer (Fig. 3B). This supports the strategic location and role of these two waters in CA flexibility and highlights a strategic role for H196 in FIV CA flexibility.

Analyzing coevolved residues using the Gremlin pseudolikelihood method, which previously predicted protein structures based on coevolved distance restraints^5^, revealed a correlation probability of ~1.0 for the adjacent pairs within HIV-1 F/Y/F and FIV Y/F/L supporting our structural-based correlation analysis (Fig. 3C). Likewise, a correlation between the FIV H196 (or HIV A204), at the trimeric interface (Fig. 3B), and the covariant FIV E200 residue (G208 in HIV) of the hook-like loop, was successfully detected (Fig. 3C). However, a correlation between the S/K pair, which was revealed in our FIV CA^CTD^ structural analysis, was poorly predicted with 0.37 probability in FIV and missed for HIV-1 (Fig. 3C), emphasizing the power of structural analysis in revealing spatially coupled correlations.

Here we provide a simple demonstration of the power of interspecies structural analysis in elucidating functional coupling of co-evolutionarily and spatially correlated pairs of substitutions, which when otherwise uncoupled and individually assessed can mistakenly implicate fragility and propose misleading hot-spots for therapeutic targeting. Structural comparison of the FIV and HIV-1 CA reveals the mechanistic basis of functional coupling of coevolved sets that spatially cooperate to preserve a viable structure, protecting the virus from assumed genetic fragility. The ability to circumvent deleterious effects of single amino acid substitutions by cooperative secondary substitutions allows mutational flexibility that may afford viruses an important survival advantage. This mechanism provides the virus with flexibility to exploit alternative but functionally equivalent patterns when the default ones are impaired by mutations, especially during the acquisition of resistance to antiviral therapeutics and cellular restrictions. The accessibility of such hidden escape mechanisms to particular viruses can be uncovered by identifying distinct but functionally equivalent interactions naturally coevolved in relative viruses. Intrinsic differences in the different selective pressures of cell culture and whole organisms underscore the need for an adequate animal model to evaluate potential risks from newly emerging resistant virus strains, and in this regard, the ability to investigate FIV coevolution in its natural host offers a unique opportunity.

## Methods

### Protein Expression and Purification

Capsid (CA) C-terminal domain (CTD, residues 143–211) of Feline immunodeficiency virus (FIV) Petaluma strain was subcloned into pET28b (Novagen) with a thrombin-cleavable His-tag at the N-terminus.

ArcticExpress cells (Agilent Technologies Genomics) containing the CA^CTD^ construct were grown to an O.D_600_ of 0.6 (LB media, 37 °C), induced with 0.5 mM IPTG and grown for a further ~20 hours at 16 °C. Harvested cells were resuspended in lysis buffer (50 mM potassium phosphate buffer pH7.5, 5 mM Imidazole, 5 mM β-mercaptoethanol and 500 mM NaCl), disrupted and the His-tagged proteins from the supernatant were captured using HisPur cobalt resin (Pierce) according to the manufacturer instructions. Protein eluted in 150 mM Immidazole was dialyzed to 20 mM HEPES pH 7.4, 1 mM DTT, and 150 mM NaCl. Thrombin (2 units/mg, Novagen) was used to cleave the His-tag (4 °C, overnight), and was subsequently removed using benzamidine sepharose resin (GE Healthcare Life Sciences). FIV CA^CTD^ protein was further purified using Superdex-200 size exclusion column (GE Healthcare Life Sciences) in dialysis buffer.

### Protein Crystallization, Data Collection and Structure Determination

Purified FIV CA^CTD^ (~7 mg/ml) was mixed with an equal volume of crystal screen well buffer and crystals were grown (20 °C) using the sitting-drop vapor-diffusion method. Diffraction quality crystals were readily obtained in the B7 condition of Index (Hampton Research) containing 56 mM Sodium phosphate monobasic monohydrate and 1.344 M potassium phosphate dibasic (1.4 M buffer at pH 8.2). Prior to data collection the crystal was flash frozen in the B7 condition of Index (Hampton Research) supplemented with 20% (v/v) glycerol.

Diffraction data was collected on a Rigaku FR-X rotating anode generator coupled to VariMax-Cu/Cr DW confocal optics and an R-AXIS HTC image plate detector, and processed using HKL3000^35^. The structure was solved by molecular replacement using Phaser^36^ and 3DTJ^33^ as a search model. The resulting model was fitted to the electron densities using COOT^37^ and refined, in CCP4 suite^38^, using REFMAC5^39^. Table 1 summarizes data collection and refinement statistics. Figures presenting structures, as well as structural superposition, were prepared using PyMOL Molecular Graphics System (Schrödinger, LLC).

## Additional Information

**Accession numbers**: Atomic coordinates and structure factors were deposited in the Protein Data Bank with the accession number 5DCK.

**How to cite this article**: Khwaja, A. *et al*. Structure of FIV capsid C-terminal domain demonstrates lentiviral evasion of genetic fragility by coevolved substitutions. *Sci. Rep.* **6**, 24957; doi: 10.1038/srep24957 (2016).

## Figures and Tables

**Table 1:** Data collection and refinement statistics.

	FIV CA CTD
Data collection	
Space group	P 6_1_ 2 2
Mol/ASU Cell dimensions	2
*a, b, c* (Å)	64.19, 64.19, 178.56
α,β,γ (°)	90.0, 90.0, 120.0
Resolution (Å)	40.63–2.29 (2.37–2.29)*
* R*_sym_ or *R*_merge_	0.108 (0.529)
* I*/σ*I*	17.91 (2.92)
Completeness (%)	99.71 (96.96)
Redundancy	16.33 (6.44)
Refinement	
Resolution (Å)	2.29
No. unique reflections	10456 (988)
* R*_work_ / *R*_free_ (5% test set)	0.235 (0.241)/0.287 (0.353)
No. atoms	
Protein	1141
Water	98
*B*-factors	
Protein	42.4
Water	41.0
R.m.s. deviations	
Bond lengths (Å)	0.015
Bond angles (°)	1.52
Ramachandran (%)	
Favored	97
Outliers	1.4
PDB code	5DCK

^*^Number in parentheses is for highest resolution shell.
